# Evaluating stress and it associated factors in mothers of preterm infants in NICU: a cross-sectional study

**DOI:** 10.3389/fgwh.2025.1570808

**Published:** 2025-09-25

**Authors:** Halyna Pavlyshyn, Iryna Sarapuk

**Affiliations:** Department of Pediatrics No 2, I. Horbachevsky Ternopil National Medical University, Ternopil, Ukraine

**Keywords:** maternal stress, Parental Stressor Scale: neonatal intensive care unit (PSS: NICU) questionnaire, neonatal intensive care unit, preterm infants, gestational age

## Abstract

**Introduction:**

Providing care of preterm infants in the NICU is a significant source of psychological and emotional stress for both infants and parents. The purpose of the study was to investigate the relationship between gestational age (GA) and maternal stress levels in the neonatal intensive care unit (NICU) setting and to identify additional demographic and clinical factors that may contribute to or exacerbate maternal stress during the infant's NICU hospitalization.

**Materials and methods:**

This observational, cross-sectional prospective study included 247 mothers of preterm infants. Maternal stress and worry were assessed using the PSS: NICU questionnaires. PSS: NICU items were organized into three subscales: Sights and Sounds (S1), Infant Appearance and Behavior (S2), and Parental Role Alteration (S3).

**Results:**

Moderate/high NICU-related stress among mothers of preterm infants was more common when infants were born before 32 weeks (*p* = 0.031), in cases of neonatal seizures (*p* = 0.027), sepsis (*p* = 0.032), and invasive ventilation (*p* = 0.027). Logistic regression showed that gestational age under 32 weeks and neonatal seizures significantly increased the risk of moderate/high maternal stress (OR = 2.90, 95% CI: 1.26–6.68, *p* = 0.012; OR = 9.83, 95% CI: 1.19–80.80, *p* = 0.033, respectively).

**Conclusion:**

NICU-related stress among mothers of preterm infants significantly associated with gestational age below 32 weeks and exacerbated by neonatal seizures. These factors can help identify mothers at high risk who may need focused support in the NICU, considering both the infant's condition and the mother's psychological well-being.

## Introduction

The clinical care and management of preterm infants in the neonatal intensive care unit (NICU) represent a considerable psychological and emotional burden for both the neonates and their parents (1, 2). NICU-related parental stress is a worldwide healthcare issue, both in high-income and low–middle-income countries (3, 4). Preterm births are often unexpected and can lead to concerns about the health and well-being of the infant (5). Uncertainty regarding the infant's survival, future health status, and quality of life, combined with a perceived loss of control over the situation and the necessity for physical and emotional separation, constitute significant factors contributing to parental stress, anxiety, worry, fear, and deterioration of mental well-being (6). Parents may also be concerned about the threat to the child's life, the unstable condition of the infant with numerous medical equipment, his/her suffering, and pain. Parents may feel anxiety due to the alteration of the parental role in the NICU: the inability to freely contact the baby, independently feed, change clothes and fully care for the child (7).

Currently, NICU-related maternal stress is insufficiently studied in Ukrainian research. In routine neonatal care, there are no standardized methods to assess the psychological condition of parents, especially mothers. This leads to a lack of timely and adequate psychological support. Factors influencing or exacerbating NICU-related maternal stress have not been sufficiently studied.

Previous international studies have shown that demographic and clinical characteristics of parents and infants influence parental emotional responses and stress during preterm infants' NICU stay (8, 9). Studies have shown that a mother's stress and negative emotions are influenced by her education, socioeconomic status, family relationships, and mental health (10, 11).

Recent studies confirm a significant association between gestational age and maternal stress levels: lower gestational age is linked to higher maternal stress and greater uncertainty about the infant's condition (12–15). In the same time, Woodward L. et al. showed that gestational age and birth weight did not correlate with the maternal stress (16). Similarly, Kraft et al. demonstrated that maternal anxiety was not associated with gestational age (17).

Due to limited data in the national context and conflicting findings from international studies, there is a need for an in-depth analysis of this issue among mothers of preterm infants in Ukraine. Gaining a deeper understanding of these factors can assist healthcare professionals in recognizing key areas for targeted interventions aimed at reducing parental stress and promoting better outcomes for both mothers and their infants (18).

Based on the above-mentioned literature review and existing research gaps, the following hypotheses were formulated for this study: (1) Lower gestational age of the newborn is associated with higher levels of maternal stress during the infant's stay in the neonatal intensive care unit (NICU); (2) Additional maternal factors (e.g., maternal age, education, employment status) and infants clinical characteristics (e.g., birth weight, morbidity) significantly influence NICU-related maternal stress levels.

**The purpose of the study** was to investigate the relationship between gestational age and maternal stress levels in the neonatal intensive care unit (NICU) setting and to identify additional demographic and clinical factors that may contribute to or exacerbate maternal stress during the infant's NICU hospitalization.

## Materials and methods

### Enrollment process and study design

This observational, cross-sectional prospective study was conducted at five regional perinatal centers in Ukraine. Each center is a level III NICU with 6–12 beds, serving an average annual birth cohort of 3,000–5,000 infants. All participating NICUs adhered to the same national neonatal care standards and demonstrated comparable organizational characteristics, including the staff-to-patient ratios, availability of medical technologies, and similar annual rates of NICU admissions. Therefore, the centers were considered homogeneous with respect to standard of care and infrastructure.

To ensure data uniformity, all variables, including respiratory distress syndrome (RDS), sepsis, necrotizing enterocolitis (NEC), and intraventricular hemorrhage (IVH), were defined according to standardized clinical criteria agreed upon by all perinatal centers participating in the study. Specifically, RDS was diagnosed based on clinical and radiological signs; sepsis was classified as either proven (positive bacterial culture) or clinical (based on symptoms and laboratory findings); NEC was defined according to the modified Bell's criteria (all stages); IVH was diagnosed and graded using cranial ultrasound findings.

***The inclusion criteria***: preterm delivery, infant's NICU-stay for at least 3 days, maternal visiting infant at the NICU at least 3 times and willingness to participate in the study with signed informed consent. ***Exclusion criteria***: history of maternal alcoholism or usage of illicit drugs, genetic disorder and/or major congenital malformations in infants.

Data on maternal and infant characteristics were obtained from medical records and supplemented by maternal history collection conducted during the infant's stay in the NICU.

### Maternal stress and worry index assessment

Maternal stress was assessed using the questionnaire Parental Stressor Scale: NICU (PSS: NICU). PSS: NICU included 26 items that were distributed in subscales: Sights and Sounds of NICU—subscale 1 (S1)—5 items, Infant Appearance and Behavior—S2—14 items, and Parental Role Alteration—S3—7 items. On a Likert scale, parents were asked to rate how stressful the experience described in each item was for them on a scale of 1 to 5. A score of 1 indicated that the factor did not cause any stress, and 5 indicated that it was extremely stressful. No experience (NE) corresponded to a situation that the mother had not experienced before. Moderate/high stress were defined as a PSS: NICU score greater than 3, with moderate stress corresponding to 3.0–3.9 and high stress to 4.0–4.9 (19).

The Worry Index questionnaire was used to measure the degree to which a mother worries about her preterm infant, his/her risk for problems. It included seven areas: medical problems, growth and development alterations, disorders, rehospitalization, sleeping and eating disorders. Items are rated on a 5-point scale, from “not at all” to “very much” (20).

Before the study began, permission to translate the questionnaires and the right to use them were obtained from the author, Prof. Margaret S. Miles, RN, PhD, FAAN Emeritus Professor, School of Nursing, The University of North Carolina. The questionnaire was translated into Ukrainian language following standard forward and backward translation procedures. Additionally, linguistic validation was conducted to ensure clarity and cultural relevance.

Cronbach's alpha coefficients for the Ukrainian version of PSS: NICU indicated good consistency for each subscale (for S1- 0.81; S2—0.94; S3—0.90), as well as the entire scale (0.95); for the Ukrainian version of Worry Index—0.92.

Maternal questionnaires were administered during the infant's hospitalization in the neonatal intensive care unit, specifically between days 3 and 7 of the NICU stay. The questionnaire was administered in person by trained healthcare staff or research assistants (depending on the center), who had received standardized instructions to ensure consistency in data collection across all sites. Mothers were given adequate time and privacy to complete the questionnaire and were offered support if any clarification was needed.

All computations were performed using StatSoft STATISTICA Version 13 (Tulsa, OK). Normality was assessed with the Shapiro–Wilk test. Quantitative data, including PSS: NICU and Worry Index scores were presented as mean (M) and standard deviation (SD). For qualitative parameters, absolute and relative frequencies (percentage) were presented. Independent samples t-test was used for comparisons between two groups, and one-way ANOVA was used for comparisons among more than two groups. Correlations were analysed using Pearson's correlation coefficient. Significance was assumed at *p* < 0.05. Proportions were compared between the two groups using the two-tailed Fisher exact test. Multiple regression was conducted to find significant predictors of moderate/high NICU-related maternal stress.

The required sample size was calculated using G*Power for a two-tailed t-test comparing two independent groups. An *a priori* sample size estimation, performed prior to the study, indicated that to detect an effect size of d = 0.5 with a statistical power of 0.95 at a significance level of α = 0.05, each group would require 105 participants. A *post hoc* power analysis based on the observed means yielded an effect size of d = 0.35. For the actual sample sizes (n₁ = 146, n_2_ = 101), the achieved statistical power was estimated at 0.776.

## Results

### Maternal and infant clinical characteristics of the study groups

There were 264 eligible mothers of preterm infants, with 247 ultimately recruited. Seven mothers did not meet the inclusion criteria, and ten mothers declined to participate and did not sign the informed consent. The distribution across the five centers was as follows: Center I—83 mothers (33.6%), Center II—31 mothers (12.6%), Center III—46 mothers (18.6%), Center IV—39 mothers (15.8%), and Center V—48 mothers (19.4%). Median maternal age was 30.0 [25.0; 34.0] years. Fifty-four (21.9%) mothers were older than 35 years. Maternal social background and history (educational level, employment status, mode of delivery, family history etc.) of the study group are presented in Table 1.

**Table 1:** Maternal and infants’ clinical characteristics of the study groups.

Indicator	Statistical indicator	Preterm infants with GA <32 weeks, *n* = 146
Maternal characteristics		
Educational level		
**–** higher education	*n* (%)	130 (52.6)
*–* secondary and incomplete secondary education		117 (47.4)
Employment status		
– employed	*n* (%)	135 (54.7)
– unemployed		112 (45.3)
Presence of depression episodes before childbirth		
– Yes	*n* (%)	54 (21.9%)
– No		193 (78.1%)
Parity		
– 1	*n* (%)	102 (41.3)
– ≥2		145 (58.7)
Delivery mode		
– vaginal delivery	*n* (%)	92 (37.2)
– caesarean section		155 (62.8)
Infant's characteristics		
Birth weight, grams	Me [Uq; Lq]	1580.00 [1200.00; 1950.00]
Birth length, cm	Me [Uq; Lq]	41.00 [37.00; 43.00]
Apgar score at 1st min	Me [Uq; Lq]	7.00 [5.00; 7.00]
Apgar score at 5th min	Me [Uq; Lq]	7.00 [7.00; 7.00]
Respiratory distress syndrom	*n* (%)	150 (60.7)
Duration of NICU treatment, days	Me [Uq; Lq]	9.0 [5.0; 16.0]
Surfactant replacement therapy	*n* (%)	79 (32.0)
Neonatal sepsis (infection)	*n* (%)	102 (41.3)
Invasive ventilation	*n* (%)	88 (35.6)
Duration of invasive ventilation, days	Me [Uq; Lq]	9.0 [6.0; 15.0]
Intraventricular hemorrhage	*n* (%)	57 (23.1)
Neonatal seizures	*n* (%)	29 (11.7)
Feeding with maternal milk (or mixed)		
	*n* (%)	103 (41.7)
Formula feeding		144 (58.3%)

There were 146 mothers of infants with GA <32 weeks (extremely and very preterm infants) and 101 mothers of infants with GA > 32 weeks (moderately and late preterm infants). The average GA of infants was 32.0 [30.0; 33.0] weeks. There were 143 (57.9%) boys and 66 (42.1%) girls. Anthropometric indicators (birth weight, birth length), Apgar score and clinical characteristics of infants are presented in Table 1.

### Associated factors of maternal NICU-related stress in mothers of preterm neonates

Maternal total PSS: NICU score was 3.36 ± 0.74. Scores by center were as follows: Center I—3.28 ± 0.85, Center II—3.41 ± 0.71, Center III—3.51 ± 0.69, Center IV—3.28 ± 0.72, and Center V—3.36 ± 0.61. There were no significant differences between centers, F (4, 242) = 0.81, *p* = 0.52, indicating that maternal NICU-related stress levels were similar across all centers. Consequently, all subsequent analyses were performed on the combined sample of mothers.

A total of 178 mothers reported moderate/high stress (54 mothers experienced high stress with a PSS: NICU score above 4, and 124 mothers experienced moderate stress with a score above 3).

**Figure 1:**
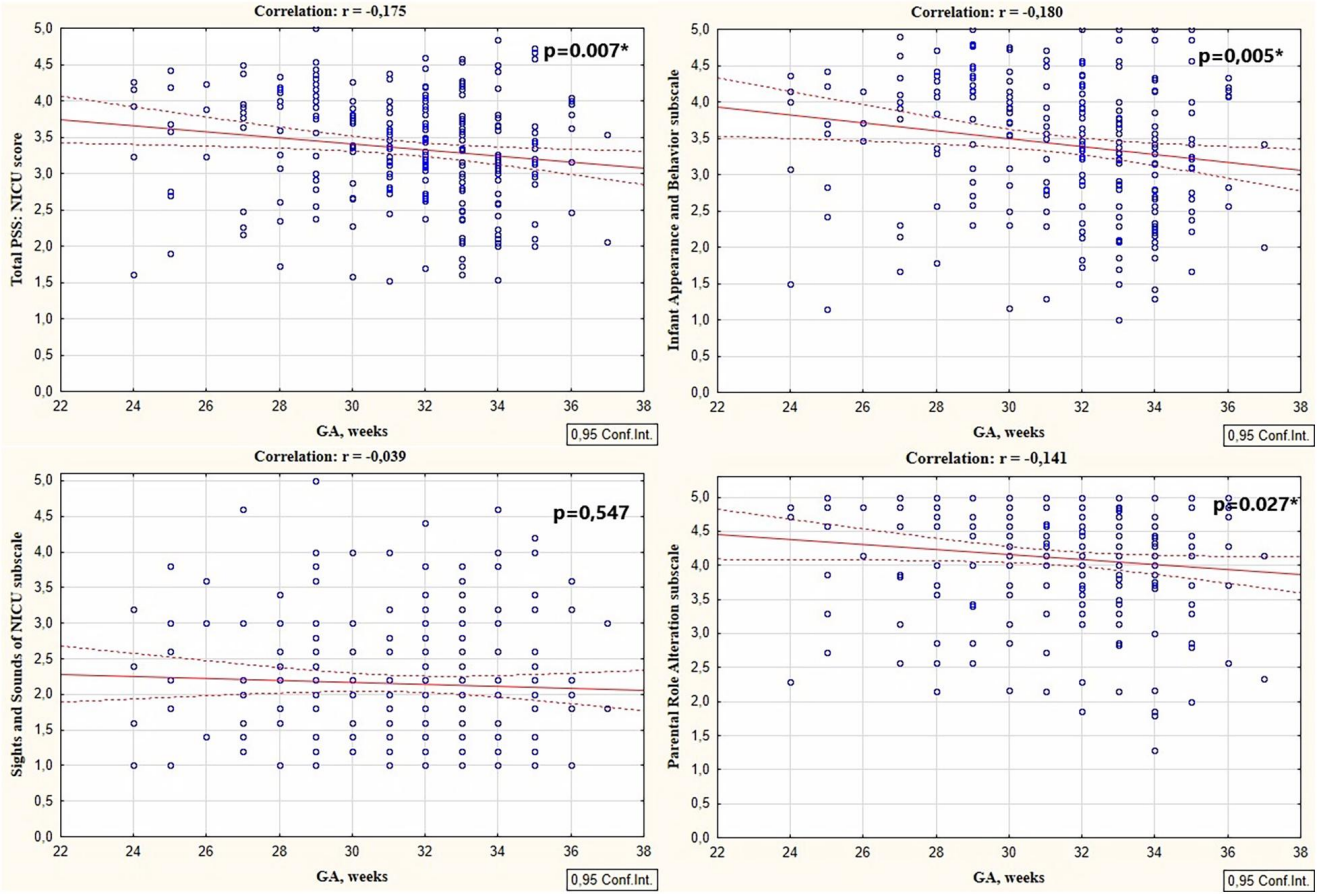
Correlations between total PSS: NICU scores, sights and sounds of NICU subscale, infant appearance and behavior subscale, and parental role alteration subscales scores with the GA.

Univariate analysis was first performed to explore the associations between maternal stress and individual maternal and infant factors. NICU-related stress was significantly higher in mothers of preterm neonates delivered before 32 weeks of gestation compared to those who delivered infants after 32 weeks (3.46 ± 0.70 vs. 3.20 ± 0.77, *p* = 0.005). Parental Role Alteration was the most stressful for mothers of both GA groups (4.20 ± 0.79 and 3.98 ± 0.89) followed by Infant Appearance and Behavior (3.58 ± 0.89 and 3.20 ± 0.92) and Sights and Sounds subscales (2.17 ± 0.89 and 2.15 ± 0.93). Significant difference in stress level associated with Parental Role Alteration and Infant Appearance and Behavior in mothers of two age groups was revealed (*p* < 0.001 and *p* = 0.045 respectively). Stress associated with Sights and Sounds in the NICU did not differ depending on GA (*p* = 0.883). The Worry Index was 27.41 ± 7.15. It was significantly higher in mothers of preterm infants with GA < 32 weeks compared to those whose infants had GA > 32 weeks (28.26 ± 7.11 vs. 26.14 ± 7.07, *p* = 0.023). Total PSS: NICU, its subscales (S2 and S3) scores and Worry index correlated negatively with the GA, Figure 1.

Maternal stress was associated with the maternal factors and infant's health status and morbidity. Sights and Sounds of NICU subscale scores were higher in mothers who had any episodes of depression before the delivery (*p* = 0.013). Total PSS: NICU and Infant Appearance and Behavior scores were significantly associated with birth weight (*p* = 0.031 and *p* = 0.004, respectively), Table 2.

**Table 2:** PSS: NICU scores in mothers of preterm infants in relation to the maternal and neonatal factors.

Variable	Total PSS: NICU score Mean ± SD	Sights and sounds (S1) score Mean ± SD	Infant appearance and behavior (S2) score Mean ± SD	Parenteral role alteration (S3) score Mean ± SD	Worry index Mean ± SD
Maternal characteristics					
Maternal age					
**–** ≤35 years	3.35 ± 0.73	2.16 ± 0.87	3.41 ± 0.93	4.11 ± 0.83	27.04 ± 7.20
– >35 years	3.36 ± 0.78	2.13 ± 1.02	3.43 ± 0.92	4.08 ± 0.89	28.69 ± 6.94
	*0*.*948*	*0*.*837*	*0*.*875*	*0*.*781*	*0*.*141*
Educational level					
**–** higher education	3.35 ± 0.82	2.22 ± 0.93	3.40 ± 0.99	4.08 ± 0.91	26.24 ± 7.50
*–* secondary and incomplete secondary education	3.36 ± 0.65	2.09 ± 0.88	3.45 ± 0.84	4.14 ± 0.75	28.72 ± 6.53
*p-value*	*0*.*970*	*0*.*228*	*0*.*638*	*0*.*542*	***0***.007*
Employment status					
**–** employed	3.37 ± 0.75	2.20 ± 0.96	3.46 ± 0.91	4.06 ± 0.88	26.91 ± 7.33
– unemployed	3.34 ± 0.73	2.11 ± 0.84	3.38 ± 0.95	4.17 ± 0.78	28.02 ± 6.91
*p-value*	*0*.*724*	*0*.*483*	*0*.*481*	*0*.*266*	*0*.*233*
Presence of depression episodes before childbirth					
**–** Yes	3.51 ± 0.76	2.43 ± 0.96	3.57 ± 0.88	4.14 ± 0.92	29.56 ± 5.88
**–** No	3.31 ± 0.73	2.08 ± 0.88	3.38 ± 0.94	4.10 ± 0.82	26.81 ± 7.37
*p-value*	*0*.*087*	***0***.013*	*0*.*175*	*0*.*737*	***0***.014*
Parity					
– 1	3.32 ± 0.80	2.16 ± 0.96	3.41 ± 0.96	4.02 ± 0.92	27.50 ± 7.25
– ≥2	3.42 ± 0.69	2.16 ± 0.89	3.49 ± 0.89	4.19 ± 0.77	27.93 ± 6.89
*p-value*	*0*.*354*	*0*.*992*	*0*.*578*	*0*.*137*	0.665
Delivery mode					
**–** vaginal delivery	3.41 ± 0.70	2.18 ± 0.90	3.53 ± 0.84	4.10 ± 0.86	26.39 ± 7.87
– caesarean section	3.36 ± 0.76	2.15 ± 0.92	3.42 ± 0.96	4.14 ± 0.83	28.50 ± 6.41
*p-value*	*0*.*611*	*0*.*757*	*0*.*388*	*0*.*769*	***0***.032*
Breast milk expression by the mother					
– Yes	3.39 ± 0.71	2.23 ± 0.96	3.49 ± 0.82	4.07 ± 0.84	27.17 ± 6.99
– No	3.31 ± 0.78	2.07 ± 0.83	3.35 ± 1.04	4.15 ± 0.84	27.71 ± 7.37
	*0*.*415*	*0*.*151*	*0*.*236*	*0*.*474*	*0*.*559*
Infants’ characteristics					
Gestational age					
– ≤32 weeks	3.46 ± 0.70	2.17 ± 0.89	3.58 ± 0.89	4.20 ± 0.79	28.27 ± 7.11
– >32 weeks	3.20 ± 0.77	2.15 ± 0.93	3.19 ± 0.92	3.98 ± 0.89	26.14 ± 7.07
	***0***.005*	*0*.*883*	***0***.001*	*0*.*046*	***0***.024*
Birth weight					
**–** <1,500 g	3.49 ± 0.73	2.14 ± 0.83	3.64 ± 0.93	4.24 ± 0.79	28.72 ± 6.83
– ≥1,500 g	3.28 ± 0.72	2.16 ± 0.96	3.30 ± 0.86	4.03 ± 0.87	26.82 ± 7.25
	***0***.031*	*0*.*869*	***0***.004*	*0*.*064*	***0***.049*
Respiratory distress syndrome					
**–** Yes	3.41 ± 0.74	2.10 ± 0.88	3.52 ± 0.92	4.16 ± 0.85	27.63 ± 7.20
– No	3.33 ± 0.73	2.29 ± 0.96	3.35 ± 0.89	4.05 ± 0.81	27.97 ± 6.71
*p-value*	*0*.*474*	*0*.*129*	*0*.*179*	*0*.*317*	*0*.*727*
Neonatal sepsis					
**–** Yes	3.49 ± 0.72	2.17 ± 0.89	3.62 ± 0.86	4.21 ± 0.80	28.09 ± 6.62
– No	3.30 ± 0.74	2.16 ± 0.93	3.34 ± 0.92	4.06 ± 0.86	27.48 ± 7.32
*p-value*	*0*.*062*	*0*.*970*	***0***.021*	*0*.*163*	*0*.*523*
Intraventricular hemorrhage					
**–** Yes	3.48 ± 0.73	2.21 ± 0.86	3.56 ± 0.93	4.23 ± 0.80	27.57 ± 8.29
– No	3.35 ± 0.74	2.16 ± 0.93	3.43 ± 0.90	4.08 ± 0.85	27.86 ± 6.54
*p-value*	*0*.*267*	*0*.*696*	*0*.*325*	*0*.*275*	*0*.*798*
Neonatal seizures (convulsive syndrome)					
**–** Yes	3.75 ± 0.49	2.16 ± 0.67	3.90 ± 0.67	4.61 ± 0.39	29.42 ± 5.87
– No	3.33 ± 0.75	2.17 ± 0.94	3.40 ± 0.93	4.06 ± 0.86	27.55 ± 7.13
*p-value*	***0***.005*	*0*.*996*	***0***.006*	***0***.001*	*0*.*218*
Invasive ventilation					
**–** Yes	3.53 ± 0.73	2.31 ± 0.94	3.66 ± 0.86	4.17 ± 0.80	28.86 ± 6.27
– No	3.29 ± 0.73	2.08 ± 0.89	3.34 ± 0.93	4.10 ± 0.86	27.07 ± 7.38
*p-value*	***0***.022*	*0*.*055*	***0***.012*	*0*.*523*	*0*.*066*
Type of feeding:					
**–** Breastfeeding and mixed feeding	3.40 ± 0.69	2.28 ± 0.96	3.53 ± 0.81	4.04 ± 0.84	27.94 ± 6.77
– Formula feeding	3.37 ± 0.77	2.09 ± 0.87	3.42 ± 0.98	4.19 ± 0.83	27.62 ± 7.20
*p-value*	*0*.*712*	*0*.*110*	*0*.*373*	*0*.*176*	*0*.*734*

The bold values indicate statistically significant results.

Neonatal seizures were the cause of an increase in total PSS: NICU (*p* = 0.005), Infant Appearance and Behavior (*p* = 0.006), and Parental Role Alteration subscales (*p* = 0.001); neonatal infection caused a higher Infant Appearance and Behavior subscale scores (*p* = 0.021). Maternal stress (total PSS: NICU and Infant Appearance and Behavior scores), was significantly associated with invasive ventilation in infant (*p* = 0.022 and *p* = 0.012, respectively), Table 2.

Worry Index was associated with the maternal education level (*p* = 0.007), mode of delivery (*p* = 0.032), presence of depression episodes before childbirth (0.014), and birth weight of infant (*p* = 0.049), Table 2.

Moderate/high stress levels were significantly more prevalent among mothers who delivered before 32 weeks' gestation compared with those who delivered after 32 weeks (*p* = 0.031). Moderate/high maternal stress was significantly more prevalent among mothers of infants with neonatal seizures (*p* = 0.027) and neonatal sepsis (*p* = 0.032) compared with mothers of infants without these conditions. Mothers of infants who required invasive ventilation were significantly more likely to report moderate/high maternal stress levels compared with mothers of infants who did not require invasive ventilation (*p* = 0.027). Depending on other maternal and infant factors, there were no significant differences in the proportion of mothers with moderate/high vs. low stress levels, Table 3.

**Table 3:** Factors associated with moderate/high levels of NICU-related maternal stress, identified by univariate analysis.

Variables	Moderate/high NICU-related maternal stress (≥3 points)	Low NICU-related maternal stress (<3 points)	*р*
Maternal age:			
– ≤35 years	41 (75.9%)	13 (24.1%)	0.607
– >35 years	137 (71.0%)	56 (29.0%)	
Educational level:			
– higher education	90 (69.2%)	40 (30.8%)	0.322
– secondary and incomplete secondary education	88 (75.2%)	29 (24.8%)	
Employment status:			
– employed	99 (73.3%)	36 (26.7%)	0.670
– unemployed	79 (70.5%)	33 (29.5%)	
Presence of depression episodes before childbirth:			
– Yes	43 (79.6%)	11 (20.4%)	0.175
– No	135 (69.9%)	58 (30.1%)	
Parity			
– 1	71 (69.6%)	31 (30.4%)	0.476
– ≥2	107 (73.8%)	38 (26.2%)	
Delivery mode			
– vaginal delivery	69 (75.0%)	23 (25.0%)	0.466
– caesarean section	109 (70.3%)	46 (29.7%)	
Gestational age			
– ≤32 weeks	113 (77.4%)	33 (22.6%)	0.031*
– >32 weeks	65 (64.4%)	36 (35.6%)	
Birth weight			
– <1,500 g	86 (76.1%)	27 (23.9%)	0.204
– ≥1,500 g	92 (68.7%)	42 (31.3%)	
Respiratory distress syndrome			
– Yes	110 (73.3%)	40 (26.7%)	0.663
– No	68 (70.1%)	29 (29.9%)	
Neonatal sepsis			
– Yes	81 (79.4%)	21 (20.6%)	0.032*
– No	97 (66.9%)	48 (33.1%)	
Intraventricular hemorrhage			
– Yes	43 (75.4%)	14 (24.6%)	0.614
– No	135 (71.1%)	55 (28.9%)	
Neonatal seizures (convulsive syndrome)			
– Yes	26 (89.7%)	3 (10.3%)	0.027*
– No	152 (69.7%)	66 (30.3%)	
Invasive ventilation			
– Yes	71 (80.7%)	17 (19.3%)	0.027*
– No	107 (67.3%)	52 (32.7%)	
Type of feeding:			
– Breastfeeding and mixed feeding	72 (69.9%)	31 (30.1%)	0.566
– Formula feeding	106 (73.6%)	38 (26.4%)	

* Statistically significant results.

Logistic regression analysis identified statistically significant factors associated with the NICU-related maternal stress (Table 4). Gestational age and the presence of seizure syndrome in infant had a significant impact. Specifically, gestational age under 32 weeks was independently associated with a 2.9-fold increased risk of moderate/high maternal stress (OR = 2.90; 95% CI: 1.26–6.68; *p* = 0.012), while the presence of seizure syndrome increased the likelihood of moderate/high stress development nearly 10-fold (OR = 9.83; 95% CI: 1.19–80.80; *p* = 0.033).

**Table 4:** Multiple regression model for the NICU-related maternal stress prediction (PSS: NICU ≥ 3).

Variable	Estimate	SE	t	*p*-level	Odds ratio	95% CI
Const.B0	−3.62	1.25	−2.91	0.004		
Maternal age > 35 years	−0.21	0.44	−0.47	0.635	0.81	0.34–1.93
Maternal higher education	0.46	0.35	1.33	0.184	1.59	0.80–3.14
Maternal employment	−0.11	0.34	−0.34	0.732	0.88	0.45–1.75
Presence of depression episodes before childbirth	−0.64	0.44	−1.47	0.142	0.52	0.22–1.24
Parity ≥ 2	−0.22	0.35	−0.64	0.525	0.80	0.40–1.59
Vaginal delivery	−0.17	0.36	−0.48	0.626	0.84	0.41–1.69
Breast milk expression by the mother	−0.33	0.35	−0.94	0.344	0.71	0.35–1.43
Gestational age ≤32 weeks	1.07	0.42	2.52	0.012	2.90	1.26–6.68
Birth weight < 1,500 g	0.10	0.40	0.24	0.806	1.10	0.49–2.45
Respiratory distress syndrome	0.65	0.40	1.61	0.107	1.91	0.86–4.23
Neonatal sepsis	−0.38	0.37	−1.02	0.308	0.68	0.32–1.42
Intraventricular hemorrhage	0.27	0.42	0.63	0.525	1.31	0.56–3.02
Neonatal seizures (convulsive syndrome)	2.28	1.07	2.14	0.033	9.83	1.19–80.80
Invasive ventilation	−0.04	0.40	−0.12	0.905	0.95	0.43–2.10
Formula feeding	0.07	0.37	0.20	0.839	1.07	0.52–2.23

Estimate, regression coefficient; SE, standard error; *t*, test statistic; *p*-level, significance level; OR, odds ratio; 95% CI, confidence interval.

## Discussion

The primary aim of this study was to explore the relationship between gestational age and maternal stress levels in the NICU, as well as to identify additional demographic and clinical factors that may contribute to or intensify maternal stress during the infant's hospitalization.

Our study highlights gestational age as a key determinant of maternal stress in the NICU, with mothers of infants born before 32 weeks experiencing significantly higher stress levels, as confirmed by logistic regression. These results are consistent with other authors who found a significant negative correlation between gestational age and parental uncertainty in the NICU (21, 22), and recent studies along with a systematic review reported that low gestational age and very low birth weight are the most common causes of parental stress (11, 23). Tilahun et al. found that parents of preterm infants delivered between 32 and 34 weeks gestation experienced higher levels of stress during the admission of their premature infants to the NICU compared to parents whose infants were delivered between 34 and 37 weeks of gestation (5). Turner et al. stated that very premature birth and twin birth were significantly associated with higher PSS: NICU scores (24). In addition, Brunson et al., in a study including infants born at or before 32 weeks gestation, found that mothers who delivered approximately one week earlier—that is, at a lower gestational age—exhibited higher levels of stress symptoms 18 months postpartum (14). However, other studies have reported that gestational age does not significantly influence maternal NICU-related stress scores (25).

Our findings can be attributed to the fact that premature infants are at a high risk of severe morbidity, developmental disabilities and are also more susceptible to life-threatening condition due to immaturity of all organs and systems (26, 27). Long-term outcomes vary widely and are very difficult to predict, adding to the uncertainty experienced by parents early in their child's life (28). The more premature an infant is, the greater the risk of morbidity and mortality (29). Accordingly, parental worry and anxiety, leading to stress, may be higher in mothers of extremely and very preterm infants compared to moderately and late preterm neonates. The larger gestational age, the more confident and calm parents are about the lesser complications their child may encounter (5). In addition, infants born at a younger gestational age require a longer duration of NICU stay and more complex specialized medical care. As a result, there is a longer separation, sometimes the inability of full-fledged kangaroo care in the NICU, a greater inability of parents to participate in the care of the child due to the life support equipment (respirators, monitors, catheters, tubes) and numerous treatment procedures (26).

Concerning the different areas of stress, Parental Role Alteration was the most stressful for mothers regardless the gestational age followed by Infant Appearance and Behavior and Sights and Sounds subscales. This indicates that parents' relationship with the infant and perceptions of their infant's appearance are the most closely associated with their overall stress levels. This finding is in line with numerous studies, that indicated that the perceived loss of the parental role is a prominent NICU-related stressor (21, 30). Also, the results of a meta-analysis conducted by Caporali et al. showed that Parental Role Alteration was the most relevant source of stress for parents in the NICU, the stress related to the physical appearance of the infant was the second greatest source of burden for parents. When infants are admitted to the NICU, the process of physiological parental attachment can be disrupted due to the physical separation, inability to take care of child, and other unique conditions of the intensive care unit (3).

Not only gestational age but also other factors may influence the intensity of maternal stress in NICU settings. According to our second hypothesis, this study also aimed to identify additional maternal factors and infant clinical characteristics that exacerbate stress and negative experiences in mothers following preterm birth.

Our results indicate a significant impact of neonatal seizures on maternal stress levels. Logistic regression analysis showed that the presence of neonatal seizures increased the likelihood of moderate/high maternal stress nearly ten-fold. In addition to the findings from the logistic regression, higher total scores on the PSS: NICU scale, as well as higher scores on the Infant Appearance and Behavior and Parental Role Alteration subscales, were observed in mothers of infants who experienced seizures. These findings align with the study by Franck et al., which showed that at the time of discharge from the neonatal unit, over half of parents of infants with neonatal seizures experienced clinically significant anxiety symptoms, and nearly one-third had symptoms of depression (31). Furthermore, two years after birth, a considerable number of mothers continued to suffer from anxiety, depression, and post-traumatic stress symptoms (32). This highlights the long-term negative impact of neonatal seizures on the mental health of parents. Additionally, parents of children with neonatal seizures often express concerns about their child's long-term neurodevelopmental outcomes, including potential learning difficulties and other disabilities (33).

Although logistic regression analysis did not identify these factors as independent predictors, univariate analyses revealed significant associations between maternal stress and several maternal and infant clinical variables. Specifically, moderate/high maternal stress was significantly more prevalent among mothers of infants with neonatal sepsis compared to those without this condition. Additionally, neonatal infection was associated with higher scores on the Infant Appearance and Behavior subscale. These findings align with Nabwire et al., who reported that mothers of infants with neonatal sepsis exhibit elevated stress levels (34). The uncertainty regarding the infant's health trajectory, combined with the visible signs of illness such as altered appearance and behavior, likely exacerbates parental distress, as reflected in the elevated Infant Appearance and Behavior subscale scores.

We also revealed, that mothers of infants who required invasive ventilation were significantly more likely to report moderate/high levels of stress compared with mothers of non-ventilated infants. Moreover, both the total PSS: NICU score and the Infant Appearance and Behavior subscale were significantly associated with invasive ventilation. Our results are consistent with previous studies demonstrating that respiratory support in infants is a significant predictor of maternal stress (8, 35, 36). These findings indicate that the need for invasive respiratory support, which is often accompanied by visible medical equipment and altered infant appearance and behavior, constitutes a major stressor for mothers in the NICU.

### Practical implementations

The obtained results highlight the importance of systematic monitoring and support of the psycho-emotional state of mothers of preterm infants, especially those born before 32 weeks of gestation. This group of mothers is at increased risk of developing moderate to high levels of stress, particularly due to disruptions in parental role and perceptions of the infant's condition.

In clinical practice, it is important to consider that significant factors influencing NICU-related maternal stress levels include the presence of seizure syndrome in the infant. This highlights the necessity for timely identification of such risks and the provision of appropriate psychological support for mothers. Additionally, other risk factors such as previous episodes of maternal depression, infectious complications in the infant, and the need for invasive ventilation should also be taken into account.

Practical measures can include a multidisciplinary approach involving psychologists, social workers, and early intervention specialists to help create a supportive environment for mothers in the NICU. Implementing these approaches can significantly improve the maternal psycho-emotional well-being, which in turn positively affects parent–child relationships, encourages better care for the infant, and may improve long-term health outcomes for both the infant and the mother.

## Strengths and limitations of the study

The main strength of this study is that it is the first known study of NICU-related maternal stress of preterm infants in Ukraine.

The main limitation of our study is the small size of interviewed mothers that should be considered when interpreting the findings. The lack of inclusion of fathers is an additional limitation and means the study provides a less complete picture of parental stress in the NICU. Despite accounting for key confounders in our analysis, residual confounding cannot be excluded. In particular, “center” was not considered as a potential confounder in the multivariate analysis, and human factors—such as staff training on communication and coping strategies—may vary between centers even if protocols, interventions, and equipment are standardized. Unmeasured psychological, socio-economic, and individual differences in maternal stress perception, as well as important variables such as support systems and the current state of conflict were not examined. These omissions represent significant limitations that may have influenced our results. Future research should incorporate these factors to better understand parental stress in this context.

## Conclusion

NICU-related stress among mothers of preterm infants significantly associated with gestational age below 32 weeks and exacerbated by neonatal seizures. These factors can help identify mothers at high risk who may need focused support in the NICU, considering both the infant's condition and the mother's psychological well-being.

## Data Availability

The raw data supporting the conclusions of this article will be made available by the authors, without undue reservation.
